# Sequential delayed [18 F]FDG PET/CT examinations in the pharynx

**DOI:** 10.1038/s41598-020-59832-4

**Published:** 2020-02-19

**Authors:** Agata Karolina Pietrzak, Andrzej Marszalek, Joanna Kazmierska, Jolanta Kunikowska, Pawel Golusinski, Wiktoria Maria Suchorska, Marcin Michalak, Witold Cholewinski

**Affiliations:** 1 Nuclear Medicine Department, Greater Poland Cancer Centre, Garbary 15, 61-866 Poznan, Poland; 2 Electroradiology Department, Poznan University of Medical Sciences, Garbary 15, 61-866 Poznan, Poland; 3 Chair of Oncologic Pathology and Prophylaxis Poznan University of Medical Sciences and the Greater Poland Cancer Center, Garbary 15, 61-866 Poznan, Poland; 4 Nuclear Medicine Department, Medical University of Warsaw, Warsaw, Poland. Banacha 1a, block E, 02-097 Warsaw, Poland; 5 Chair, Department of Otolaryngology and Maxillofacial Surgery, University of Zielona Gora, Zyty 28, 65-046 Zielona Gora, Poland; 6 Radiobiology Department, Greater Poland Cancer Centre, Garbary 15, 61-866 Poznan, Poland; 7 Gynecologic Oncology Department Greater Poland Cancer Centre, Garbary 15, 61-866 Poznan, Poland

**Keywords:** Oncology, Cancer

## Abstract

This study aimed to evaluate the usefulness of the biphasic 2-deoxy-2-[18 F]fluoro-D-glucose positron emission tomography/computed tomography ([18 F]FDG PET/CT) examinations in terms of distinguishing benign and malignant lesions within the pharynx. 139 patients underwent sequential biphasic [18 F]FDG PET/CT examinations at 60 and 90 minutes (min) post intravenous injection (p.i.) of the [18 F]FDG. We evaluated the metabolic activity of 93 malignant lesions and 59 benign findings within pharynx as well as 70 normal blood vessels. We evaluated the maximal and mean standardized uptake value (SUVmax, SUVmean) and the retention index (RI-SUVmax). We used the receiver operating characteristics (ROC) analysis to obtain the prognostic metabolic indices cut-off which may differentiate between benign and malignant lesions. The SUVmax value cut-off at 60 and 90 min p.i. differentiating between normal and abnormal metabolic activity in the pharynx was 1.9 and 2.0, respectively. When compared benign and malignant lesions, the SUVmax on initial and delayed scans were 3.1 and 3.6, respectively. In this material, the increase of the SUVmax value over time of 1.7% suggested abnormality, while RI-SUVmax of 5.7% indicated malignant etiology. The biphasic [18 F]FDG PET/CT study protocol is useful in better stratification of normal and abnormal glucose metabolism activity in the pharynx.

## Introduction

The most common malignancy in the head and neck region is the squamous cell carcinoma (SCC)^1^ of pharynx^2,3^. The 2-deoxy-2-[F-18]fluoro-D-glucose positron emission tomography/computed tomography ([18 F]FDG PET/CT) examination is used in the head and neck cancer patients mainly for staging and follow up^3,4^. It can be an accurate method used in deciding on performing neck dissection on patients with residual disease in the neck post-primary radiochemotherapy^5,6^. The significant limitation of the technique is the non-tumor-specific [18 F]FDG property resulting in difficulties in the differential diagnosis due to glucose metabolism similarity of some non-cancerous but biologically active structures^7,8^ in standard, single-time-point (STP) examinations. The delayed (dual-time-point, DTP, biphasic) [18 F]FDG PET/CT studies might be used to increase the specificity of the method due to the possibility to evaluate the maximal and mean standardized uptake value (SUVmax, SUVmean) changes over time^8^. The most commonly used DTP protocols are at 60 and 120 minutes (min) or at 60 and 180 min post-injection (p.i.) of the [18 F]FDG. In this study, we have described the sequentially performed biphasic scanning as possibly more convenient considering patients’ comfort, radiation safety and more accessible for the technician when compared to above-mentioned protocols. We have evaluated the prognostic SUVmax and the retention index (RI-SUVmax) cut-off values, using receiver operating characteristics (ROC) analysis, which might suggest the lesions’ etiology. The DTP studies were mostly examined in the chest region while the head and neck region and the sequential protocol were not widely investigated before^9,10^. The study aimed to evaluate the utility of the subsequent delayed [18 F]FDG PET/CT examinations in determination normal and pathologic lesions within the oropharynx, nasopharynx and hypopharynx with the particular focus on the potential role of the method in increasing the specificity of the method.

## Methods

The study was performed upon received of the patients’ written informed consent and approved by the Local Bioethical Committee (Poznan University of Medical Sciences Bioethical Committee, chair: Pawel Checinski, Prof.) as the retrospective analysis based on standardly performed examinations from December 2014 to May 2017. All data has been anonymized so examined patients cannot be identified. All steps of the examinations have been performed in accordance with the Bioethical Committee guidelines and the Declaration of Helsinki.

### Epidemiological and histologic data

In this study, 139 consecutive patients (45 women, 94 men, Table 1) underwent sequential delayed [18 F]FDG PET/CT examinations. The inclusion criteria for the diagnostic procedure were as follows: suspicious findings within the pharynx and abnormal lesions within the pharynx observed on PET scan, no treatment received, histopathologic examination performed after the scanning. Patients in whom following protocol criteria were not preserved, were excluded from the analysis.

**Table 1:** Epidemiological data.

Epidemiological data					
Characteristic	SCC Oropharynx	SCC Nasopharynx	SCC Hypopharynx	Inflammatory lesions	Postoperative lesions
**Value**					
Number of patients/lesions	22/27	41/47	17/19	47/47	12/12
Number of men/women	15/7	27/14	14/3	27/20	11/1
Mean age ± S.D. [years]	59 ± 11	55 ± 15	63 ± 11	57 ± 13	62 ± 7
Range [years]	32–72	22–93	28–77	21–81	51–78

We have evaluated 152 (benign and malignant lesions) in 139 patients (Table 1). We have evaluated 70 blood vessels for the comparative purposes (normal and abnormal metabolic activity). The cohort was divided into groups considering histopathologic confirmation and the follow-up. The benign lesions group consisted of and inflammatory (histologically confirmed granulocytic infiltration) and postoperative lesions (Table 1). We have obtained 93 tumors in 80 patients – histologically examined SCC oropharynx, SCC nasopharynx, SCC hypopharynx in patients before the treatment. We have evaluated 59 benign lesions with granulocytic infiltration observed in the histologic examination in 59 patients. 70 normal structures - blood vessels (common carotid artery) have been evaluated in the examined group as normal metabolic activity level indicator (SUV value higher than within local blood vessels was considered as abnormal). All structures were assessed in the whole volume.

From the group of 139 patients, we chose randomly 70 and evaluated blood vessels SUV value levels (mean age: 62 ± 13 years, range: 21–84 years): 25 women, 45 men. When analyzed the normal, all benign and all malignant pharynx subsites, groups were well balanced in the term of age and number of delineated structures.

### The study protocol

The [18 F]FDG PET/CT imaging protocol consisted of two sequentially performed acquisitions: at 60 min^1^ (whole body, range: 58–67 min) and at 90 min (head and neck region, range: 78–95 min) p.i. of the radiopharmaceutical [18 F]FDG in activity of 3.7 megabecquerels per kilogram of body mass (MBq/kg; 0.1 millicuries per kilogram - mCi/kg), average: 303.4 MBq (8 mCi), range: 254–433 MBq (7–12 mCi) with the PET/CT scanner Gemini TF16, Philips (Cleveland, Ohio, United States of America). The time of the whole scanning did not exceed 37 min (Table 2). We have performed the initial and delayed CT scanning with comparable technical parameters: beam energy of 120 kilovoltage peak (kVp), beam amperage of 100–200 milliampers per second (mAs), tube rotation of 0.5 s, Pitch of 0.8. The PET imaging was performed with the 90 s per bed position. As a standard preparation protocol, patients fasted for at least 6 hours (h; the glucose level did not exceed 150 milligrams per deciliter - mg/dL)^11^, avoided low-temperature environment and increased physical activity for 48 h prior the study^12^.

**Table 2:** Sequential biphasic [18 F]FDG PET/CT study protocol.

Phase and area of scanning	Characteristic	Value [min]
Initial 60 min p.i.	mean start time p.i. ± S.D.*	63 ± 3
	Range p.i.	58–67
Whole body scanning: mid-thigh - skull vertex	mean scanning lenght	19 ± 2
	Range	16–21
Delayed 90 min p.i.	mean start time p.i. ± S.D.	91 ± 3
	Range p.i.	78–95
Head and neck region: skull vertex - aortic arch	mean scanning lenght	7 ± 3
	Range	5–9
Initial and delayed	mean total delay between phases	5 ± 2
	Range	4–7

*S.D. – standard deviation.

### Glucose metabolism activity markers

We have measured the metabolic indices with following equations (1), (2) and (3):^9,10,13^

1. SUVmax = maximum tissue concentration [MBq/kg]/(injected dose[MBq]/body weight [kg])
2. SUVmean = average tissue concentration [MBq/kg]/(injected dose[MBq]/body weight [kg])
3. RI-SUVmax = 100% x [(SUVmax90min p.i.–SUVmax60min p.i.)/SUVmax60min p.i.)]

### Methods of segmentation

**Figure 1:**
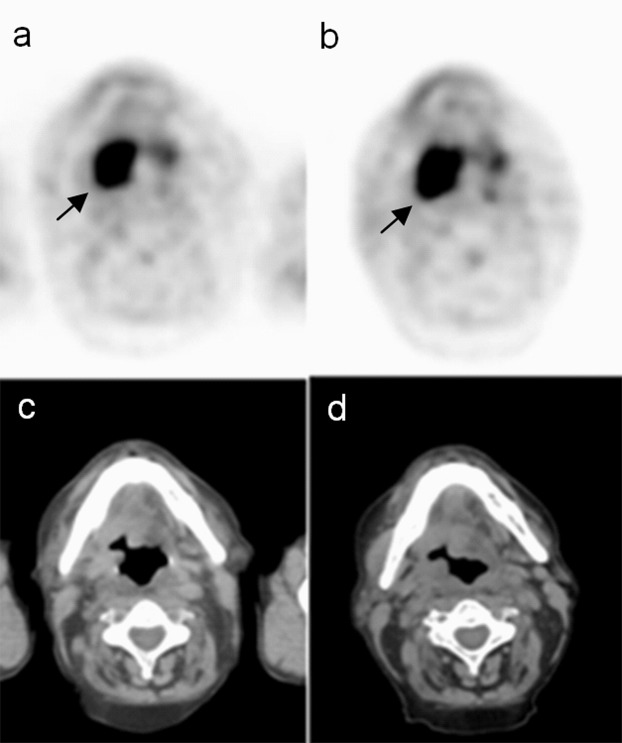
The malignant lesion within the pharynx showing increasing [18 F]FDG uptake over time. (**a**) - PET initial scans; (**b**) - PET delayed scans; (**c**) - CT initial scans; (**d**) - CT delayed scans; black arrows show the malignant mass infiltrating oropharynx.

We have used the semi-automatic method of segmentation with 50% background cut-off (Philips Fusion Viewer application) to delineate lesions within the head and neck region (Fig. 1). We reconstructed initial and delayed images using a spatial resolution of 5 mm, 3 mm, respectively.

## Results

**Figure 2:**
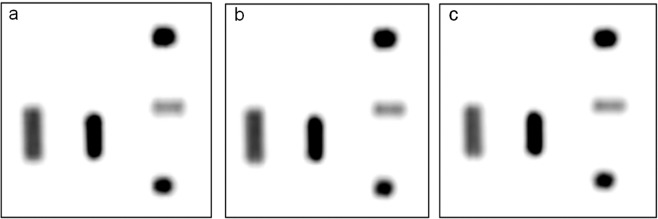
The phantom study.

We performed the phantom research to confirm that SUVmax, SUVmean values changes over time insignificantly differ when there are no biologically active process within the region of interest (ROI). We have observed no significant differences in the SUV values results between the initial and delayed acquisitions when considered glucose metabolism activity, nor when the spatial resolution has been increased^14^ (Fig. 2, Table 3).

**Table 3:** Phantom study – SUV max value measurements.

Sample volume [cm3]	Initial and delayed SUVmax values		
Image (phase; spatial resolution)	a (60 min; 5 mm)	b (90 min; 5 mm)	c (90 min; 3 mm)
2	7.7	7.7	7.7
4	2.6	2.6	2.7
10	14.0	13.9	14.0
15	3.5	3.5	3.5
20	15.6	14.4	15.5

Legend: a-initial scanning, b-delayed scanning, c-delayed scanning and increased spatial resolution (as shown on the Fig. 2).

### Statistical analyses

We have used the Statistica, Statsoft, Poland software to perform the necessary analyses. We have analyzed the distribution of the variables with the Shapiro-Wilk test. We used the t-test for dependent and independent variables, the U Mann-Whitney’s tests when compared two groups and the Kruskal-Wallis test when investigated differences of the metabolic parameters between three or more groups of diagnosis. We considered the materiality level of P < 0.05 as statistically significant in every step of the analysis. The SUVmax and SUVmean values at 60 and 90 min p.i. significantly differed from the Gaussian model within the following groups: lesions located within nasopharynx, hypopharynx, inflammation, all malignant and the non-malignant structures (P < 0.001). The variables were normally distributed within oropharyngeal cancer lesions, blood vessels, and postoperative areas. The RI-SUVmax database consisted of normally distributed variables when analyzed following groups of structures: malignant lesions localized within oropharynx (P = 0.6) and hypopharynx (P = 0.6), benign lesions: inflammation (P = 0.4), postoperative changes (P = 0.9) and all benign findings (P = 0.5). The RI-SUVmax evaluation showed that nasopharynx cancer lesions, all malignant and the blood vessels datasets significantly differed from the Gaussian distribution (P = 0.003, P < 0.001, P < 0.001, respectively). When analyzed the normally distributed variables, the average values and standard deviation were obtained. If the distribution significantly differed from the Gaussian model, the SUV value median and mode has been calculated.

### The SUVmax and SUVmean values evaluation

According to our results, the SUV value levels were the highest and significantly increased over time within malignant tumors, while within benign lesions the glucose metabolism activity levels were constant (Table 4).

**Table 4:** Glucose metabolism changes over time within analyzed lesions.

Diagnosis/Measurements	SUVmax initial and delayed	SUVmean initial and delayed	Tendency over time
SCC Oropharynx	p < 0.001	p < 0.001	increasing
SCC Nasopharynx	p < 0.001	p < 0.001	increasing
SCC Hypopharynx	p < 0.001	p < 0.001	increasing
Inflammation	p = 0.4	p = 0.3	no change^a^
Postoperative lesions	p = 0.1	p = 0.1	no change^a^
Blood vessels	p < 0.001	p < 0.001	decreasing

^a^Statistically insignificant increase of the [18 F]FDG uptake over time.

In this material, the tumors localized in the hypopharynx showed the highest glucose utilization within the malignant lesions group. When evaluated benign structures, we found that activity of inflammation and postoperative lesions were comparable. Tables 5 and 6. show the SUVmax, SUVmean values at 60 and 90 min p.i. of the [18 F]FDG biodistribution over time within five individual study groups of lesions observed in the head and neck region, excluding blood vessels (Table 5) and the consistent SUV value levels data of three main groups of analysis (blood vessels, benign and malignant lesions, Table 6).

**Table 5:** The SUVmax, SUVmean at 60 and 90 min p.i. of the [18 F]FDG within analyzed groups.

Lesion/Characteristic	SCC Oropharynx	SCC Nasopharynx	SCC Hypopharynx	Inflammatory	Postoperative
Value					
**SUVmax 60 min**					
Median	4.7	4.4	4.7	3.2	2.7
Mode/multiplety	8.8/2	2.6/3	—*	2.7/3	1.8/2
Range	2.7–8.9	2.2–14.4	2.2–14.0	2.0–7.0	1.8–3.5
**SUVmax 90 min**					
Median	6.1	4.8	5.4	3.5	2.7
Mode/multiplety	3.3/2;4.2/2	11.1/2;5.0/2	—	2.6/2;2.8/2	—
Range	2.9–10.5	2.3–16.1	2.9–14.2	1.8–6.7	1.9–3.6
**SUVmean 60 min**					
Median	3.1	2.7	3.2	2.6	2.1
Mode/multiplety	4.7/2	2.0/2	—	2.4/3	1.7/2
Range	1.6–6.9	1.3–12.9	1.7–10.4	1.5–5.8	1.4–2.6
**SUVmean 90 min**					
Median	3.4	3.1	3.6	2.7	2.1
Mode/multiplety	2.5/2;3.3/2	5.3/2	—	—	1.9/2
Range	1.6–7.5	1.5–14.4	1.9–10.6	1.4–5.0	1.5–2.9

*No mode observed.

**Table 6:** The SUVmax, SUVmean at 60 and 90 min p.i. of the [18 F]FDG within malignant, benign lesions and blood vessels.

Lesion/Characteristic	Malignant lesions	Benign lesions	Blood vessels
Values			
**SUVmax 60 min**			
Median	4.6	3.1	1.5
Mode/multiplety	2.6/3	2.7/4	1.5/5
Range	2.2–14.4	2.2–14.4	0.8–2.0
**SUVmax 90 min**			
Median	5.1	3.3	1.3
Mode/multiplety	3.1/2;4.0/2	2.6/3	1.1/4
Range	2.3–16.1	2.3–16.1	0.5–1.9
**SUVmean 60 min**			
Median	3.0	2.4	1.2
Mode/ multiplety	4.7/3	1.9/2;3.3/2;4.2/2	0.9/5;1.0/2;1.1/3
Range	1.3–12.9	1.3–12.9	0.7–1.7
**SUVmean 90 min**			
Median	3.3	2.6	1.1
Mode/ multiplety	1.9/2;3.3/2	1.5/3;1.6/2;1.7/2	0.7/4;1.0/3,1.1/3
Range	1.5–14.4	1.5–14.4	0.3–1.9

### The RI-SUVmax calculation

According to the Kruskal-Wallis test’s results, the RI-SUVmax value differences between three main groups of comparison: blood vessels, all benign and all malignant lesions, were significant with P < 0.001. The highest mean RI-SUVmax value has been observed within the SCC hypopharynx group, while the lowest in the inflammation and blood vessels groups (Table 7).

**Table 7:** The RI-SUVmax calculation.

The RI-SUVmax evaluation		
Lesions/value	mean RI-SUVmax ± S.D.	Range
SCC Oropharynx	11% ± 10%	−10% to 36%
SCC Nasopharynx	12% ± 14%	−9% to 51%
SCC Hypopharynx	17% ± 19%	−14% to 66%
Inflammation	1% ± 10%	−18% to 28%
Postoperative lesions	6% ± 10%	−13% to 24%
Blood vessels	−13% ± 12%	−53% to 2%
Benign lesions	2% ± 10%	−18% to 28%
Malignant lesions	12% ± 14%	−14% to 66%

### The prognostic SUVmax and RI-SUVmax values cut-off values

**Figure 3:**
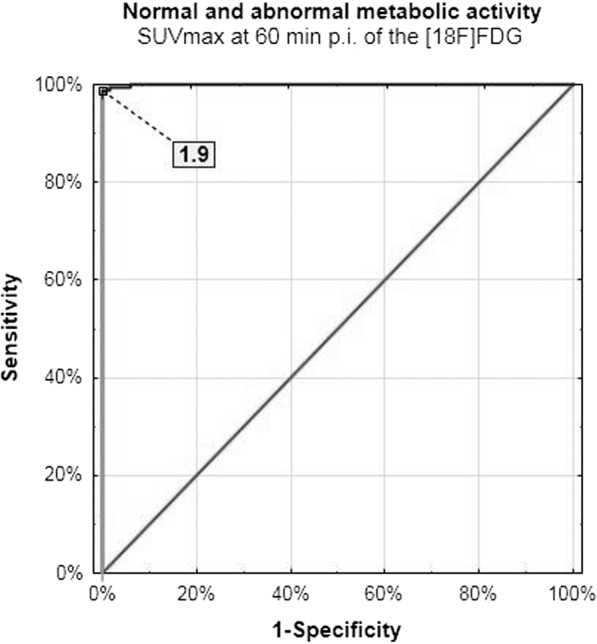
Normal and abnormal metabolic activity cut-off value – initial scanning.

**Figure 4:**
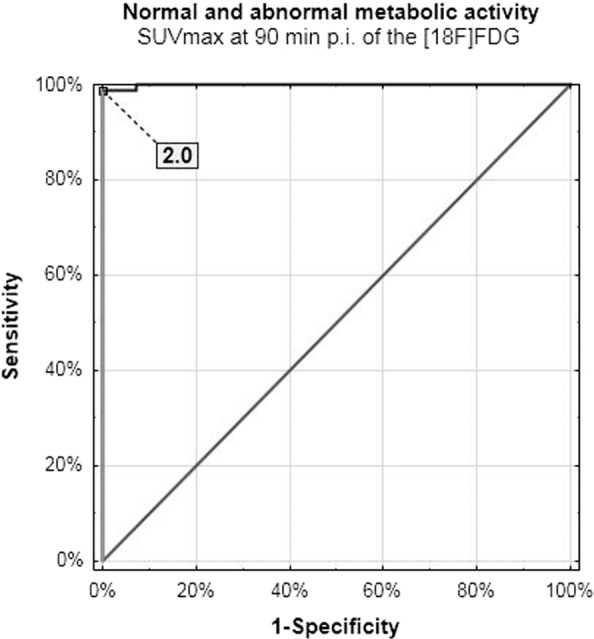
Normal and abnormal metabolic activity cut-off value – delayed scanning.

In this study, the SUVmax cut-off value differentiating normal (physiologic blood vessels) and abnormal glucose metabolism activity was increasing over time with comparable sensitivity and specificity on initial and delayed scans (Figs. 3 and 4). The ROC analysis showed that any increase of SUVmax value over time indicates abnormality within ROI.

**Figure 5:**
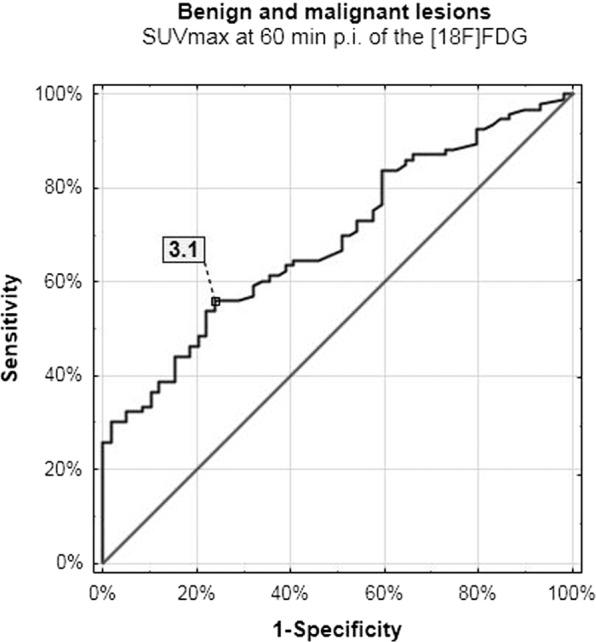
Benign and malignant lesions – initial scanning.

**Figure 6:**
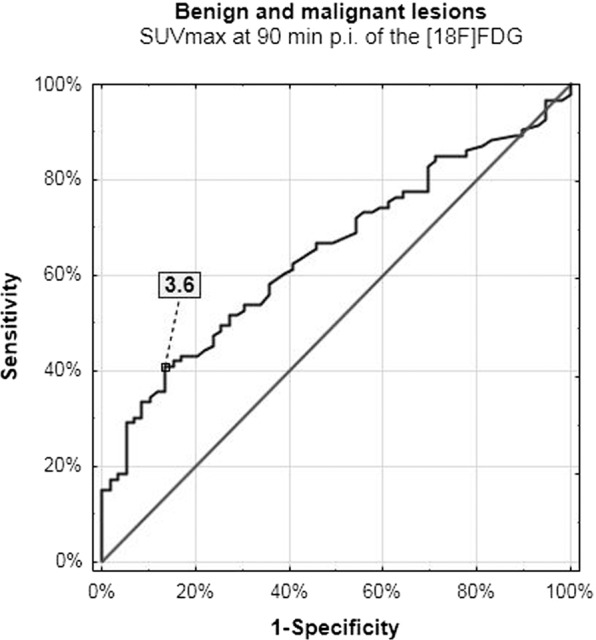
Benign and malignant lesions – delayed scanning.

The delayed phase of scanning increased the specificity of the method in distinguishing benign (inflammation, postoperative lesions) and malignant lesions of 10% when compared to the initial images (Figs. 5 and 6). In this material, the 5.7% increase in glucose utilization suggested malignant etiology.

In this material, the sequential biphasic [18 F]FDG PET/CT studies increased the specificity of the method in distinguishing lesions of different etiology within the pharynx. We have found that sequential biphasic protocol can differentiate between normal and abnormal metabolic activity with the specificity up to 100% and evaluated the reliability of the used test, which has been described as the ROC analysis report summary in Table 8.

**Table 8:** The ROC analysis results.

ROC analysis report					
Characteristics	cut-off value	sensitivity/specificity[%]	Youden Index	AUC[%]	P-value*
**Normal blood vessels and all abnormal lesions**					
SUVmax 60 min	1.9	99/100	0.99	100	<0.001
SUVmax 90 min	2.0	99/100	0.99	99	<0.001
RI-SUVmax [%]	1.7	73/99	0.7	89	<0.001
**Benign and malignant lesions**					
SUVmax 60 min	3.1	56/76	0.3	69	<0.001
SUVmax 90 min	3.6	41/86	0.3	65	<0.001
RI-SUVmax [%]	5.7	71/64	0.4	71	<0.001

*Kruskal-Wallis test’s results: differences between three groups.

## Discussion

In some cases, the similarity of metabolic properties between benign and malignant lesions make the cancer patients’ management difficult when performing standard STP [18 F]FDG PET/CT studies. Thus, the modification the diagnostic approach seems to be of value. According to the literature^13,15^, the high (≥ 2.5) and increasing SUV value over time suggests malignancy, while the constant or decreasing glucose metabolism parameters provide information that observed lesion is possibly benign (Tables 4 and 7).

The main clinical advantage in the pharynx is to indicate the primary tumor location. When the primary tumor’s volume is not sufficient enough to be evaluated with the available spatial resolution of the used method, the possibility to detect the neoplastic disease origin is difficult. Very often, only the metabolic activity increment within the ROI suggests abnormality for the nuclear medicine specialist while the morphologic image does not show a tumor but the metastatic lymph nodes occurrence. Calculating the SUV value changes over time might be helpful in post-therapeutic patients’ management to distinguish recurrent or remnant tumor and post irradiated lesion and primary tumor and tumor’s recurrence differential diagnosis, especially within the oropharynx^16,17^. It can also be helpful in patients’ monitoring or therapy planning^16–18^. The main goal of the diagnostic management is to help the surgeon to choose the area which should be histologically examined (“golden standard”), which is crucial to avoid false positive and false negative diagnostic results.

Performing DTP examinations may increase the specificity of the method in discrimination of normal and pathologic glucose metabolism activity. The sequential scanning is possibly more acceptable than the more delayed approach for patients and the radiation safety requirements as it does not prolong each patient’s stay in the nuclear medicine department. The sequential biphasic imaging does not demand a patient’s repositioning between the phases of scanning. Therefore, the whole imaging procedure is less time-consuming and easier for the operator. Avoiding repositioning can be more convenient considering the initial and delayed images comparison and the evaluation by the physician than more delayed protocol. Moreover, according to our results compared with the literature^19–21^, performing the more delayed protocol and the sequential scanning provide similar results.

The results obtained in this study were in line with the available literature describing DTP examinations, which provides information that sequential biphasic scanning can be performed as an alternative to more delayed protocols. According to our results, the SUVmax and RI-SUVmax cut-off values of 2.0 and 1.7%, respectively, can differentiate normal and abnormal glucose metabolism activity in the head and neck cancer patients when analyzed the pharyngeal subsites with sensitivity / specificity ratio of 99% / 100% (Table 8). *Houshmand et al*. show comparable results when assessed primary tumors within the liver with more delayed protocol: at 60 and 120 min p.i. of the [18 F]FDG, using the local blood vessels as the referential SUV value level (Table 6)^19^. In this material, the SUVmax and RI-SUVmax values of 3.6 and 5.7%, respectively, distinguished benign from malignant lesions (Table 7 and Table 8). Lee *et al*. and *Zhang et al*. observed comparable tendencies and sensitivity to specificity ratio when analyzed the thyroid gland pathologies and lymph nodes in the chest region with the delayed [18 F]FDG PET/CT study protocol performed at 60 and 120 min p.i. of the [18 F]FDG^20,21^.

## Conclusions

The sequential biphasic [18 F]FDG PET/CT scanning protocol can be used as an additional study which may increase the sensitivity and the specificity of the method in distinguishing benign and malignant lesions within the pharynx.

## Data Availability

The datasets analysed during the current study are available from the corresponding author on request.
